# Environmental Characteristics of Polybrominated Diphenyl Ethers in Marine System, with Emphasis on Marine Organisms and Sediments

**DOI:** 10.1155/2016/1317232

**Published:** 2016-11-24

**Authors:** Ying Zhang, Weiliang Wang, Jinming Song, Zongming Ren, Huamao Yuan, Huijun Yan, Jinpeng Zhang, Zhen Pei, Zhipeng He

**Affiliations:** ^1^Institute of Environment and Ecology, Shandong Normal University, Jinan 250014, China; ^2^College of Geography and Environment, Shandong Normal University, Jinan 250014, China; ^3^Institute of Oceanology, Chinese Academy of Sciences, Qingdao 266071, China; ^4^Fishery Design Institute of Shandong Province, Jinan 250014, China

## Abstract

Polybrominated diphenyl ethers (PBDEs), due to their widespread usage as flame retardants and their lipophilicity and persistence, have become ubiquitous in the environment. It is urgent to understand the environmental characteristics of PBDEs in marine system, but they have attracted little attention. We summarize the available data and analyze the regional distributions, controlling factors, and congener patterns of PBDEs in marine and associated environmental matrixes worldwide. Based on meta-analysis, after separating the estuarial sites from the marine sites, ignoring the extraordinary sample sites such as those located just near the point source, the PBDE concentration levels are still in the same order of magnitude from global scale. Despite Principal Component Analysis, the congener patterns of sediments are predominant with the heavy brominated congeners (BDE-209 contributing over 75% to the total load) while the biota abound with the light ones (BDE-47, BDE-99, and BDE-100 taking about 80%). The ratio between BDE-99 and BDE-100 for the lower trophic-level species often turns to be greater than 1, while for those higher species the ratio may be below 1, and some species feed mainly on the crustaceans and zooplankton seems to have a higher ratio value. The data of the PBDEs in marine system are currently limited; thus, data gaps are identified as well.

## 1. Introduction

Polybrominated diphenyl ethers (PBDEs) have been produced and applied extensively as additive brominated flame retardants (BFRs) in various consumer products, such as plastics, textiles, and electronic equipment, in recent decades [1–3], as they are not chemically bond to materials and can be easily released into environment during production, use, disposal, and recycling process. PBDEs are highly hydrophobic and bioaccumulative [4–6] and have the propensity to enter the gas phase at ambient conditions and undergo long-range atmospheric transport [7, 8]. They have been found to have a ubiquitous environmental distribution and have been measured at remote sites, like the Arctic, where they had never been used [9, 10] and are ubiquitous in the sediments [11], soil [12], air [13], and divers biotic species [14]. PBDEs exposure has become a worldwide pollution problem. These properties led to the inclusion of the penta- and octatechnical mixtures in Annex A (elimination) of the convention (SC-4/14 and SC-4/18) at the meeting of the conference of parties (COP) in May 2009 [15]. It seems to be the morning twilight to solve the environmental problem of PBDEs. However, recent publications report that, overall, legacy PBDEs are still identified at higher concentrations than emerging non-PBDE BFRs in Europe [14].

Scientists are devoted to flesh out the status of PBDE contaminations in environment. PBDEs have been the focus of numerous studies and reviews for over decades [14], including the regional distributions [16–18], exposures [19, 20], toxicities [21], and time trends [22, 23], but seldom of them have been conducted concerning the environmental characteristic in marine system. Ocean is the major sink of these pollutants, via (i) direct deposition from the atmosphere, (ii) runoff from land, (iii) direct discharge from industry or wastewater treatment [2, 24–26]. Ocean plays an important role in the fate of PBDEs via gravitational sinking of particulates into sediments and their biotransformation via biometabolism process. It is urgent to determine their distributions in marine environment, based not only on the absence of any previous data, but also on the complexity of their environmental fate and effects, coupling with bioaccumulation potential, environmental recalcitrance, and potential human and wildlife toxicities.

The object of this review is to reanalyze the published data to portray the current state of knowledge about the distributions, contents of PBDEs in marine environment worldwide, where sediments and biota are specially emphasized on. The controlling factors on the distributions of PBDEs in matrixes are also discussed here. Furthermore, congener patterns in marine and associated environmental matrixes are discussed in detail. The data for PBDEs in marine system are currently limited; thus, research recommendations and data gaps are identified and discussed as well.

## 2. Data Treatment

PBDEs are presented as mixtures of congeners, and thus the set of congeners reported in the various papers were not consistent. It is somewhat difficult to evaluate the relative degree of PBDE contaminations across various studies. As a compromise of the divergence comes from congeners chosen, several aspects were taken as considerations. We were inclined to choose (i) the major components in the three commercial products (Penta-, Octa-, and Decamixtures) (such as BDE-47, BDE-99, BDE-100, and BDE-153 in Pentamixtures and BDE-153, BDE-183, BDE-197, and BDE-207 in Octamixtures, and BDE-209 in Decamixtures [27]); (ii) those frequently reported (such as BDE-15, BDE-17, BDE-28, BDE-47, BDE-66, BDE-85, BDE-99, BDE-100, BDE-138, BDE-153, BDE-154, BDE-183, and BDE-209); (iii) excluding those playing a minor role to the total load or even generally under the determine limit ones (such as BDE-15, BDE-17, BDE-66, BDE-85, and BDE-138). Therefore, only eight congeners, BDE-28, BDE-47, BDE-99, BDE-100, BDE-153, BDE-154, BDE-183, and BDE-209 are selected for investigation due to the above considerations. The total PBDE concentrations have been normalized to the sum of the eight congeners (hereafter referred to as ∑_8_PBDEs, except when stated otherwise), to exclude the variances of the congener numbers detected.

The units are made consistent in the same matrix. For example, ng/g dry weight for sediments and ng/g lipid weight for marine organisms. ng/g wet weight was converted to lipid-adjusted concentrations, using given lipid concentrations or estimating from other published researches (as described below). Unit of the atmosphere samples is consisted on pg m^−3^.

As the data from various studies cannot be assumed to be normally distributed, in some studies one or two of the sample locations may have been easily disturbed by occasional point source inputs [28], and median value is chosen to evaluate the polluted levels of PBDEs rather than average.

In our review, the median was chosen to evaluate the polluted levels of PBDEs rather than average, because the data from various study cannot be assumed to be normally distributed; in some parts of the study one or two of the sample locations may have been easily disturbed by occasional point source inputs.

In most of the study the concentrations of congeners in some samples were not available because of being lower than detection level. Several authors treated these missing values as zero or half of the detection limit. Besides these methods, compound ratios are used in the sum to assess the concentration of these congeners. A compound ratio (CR) is the ratio between two or several compounds, as defined in

(1) $$\begin{array}{c} CR=\frac{a_{1}}{a_{1}+a_{2}}. \end{array}$$

In the formula, *a*_1_ and *a*_2_ are the concentration of compounds 1 and 2 in a sample. When using compound ratios, missing values do not affect all variables, and their affecting ratios are defined as missing. If the missing values are up to 50%, the method is not to be used anymore.

Principal Component Analysis performed by SPSS (version 22.0) ORIGIN (9.0) is used to compare the congener patterns of PBDEs in the sediment, biological, atmosphere samples, and three commercial mixtures.

## 3. Marine Environmental Levels

PBDEs released from various sources can be transported into marine system via riverine inputs and atmospheric deposition. PBDEs have high binding affinity to particles and lipids and tendency to accumulate in sediment [26, 29] and biota [30]. Since the hydrophobicity nature, PBDEs have very low water solubility [2], and only a little portion of the PBDEs exist in the water phase. Due to the lower concentrations and technical difficulty in measurement, to our knowledge, few papers have been published related with sea water [31, 32]. Here, main focuses were set on the sediment and biota matrixes.

### 3.1. Sediment

Sediment is an important sink of anthropogenic pollutants and has large impact on their distributions and transport in aquatic environment [33]. We examined most of the available published works and recalculated the published data to understand the regional distributions of PBDEs in marine sediments. Although the published data are still far more enough to describe the worldwide contamination situation of PBDEs in marine environment, unless we have no other choice as noticed in the manuscript or Table 1, most of the interesting data referring to other aquatic environment such river [11, 34] or lake [35, 36] are not included in this section, and even some of the papers reported that the data on marine sediment are not included either, if the BDE-209 (mostly the dominant congener in sediment) was not available for us [37].

As shown in Table 1, pollution level is still comparable within the data collected worldwide. When we reanalyzed the published data, separated the estuary sites from the marine sites, and ignore the extraordinary sample sites such as those located just near the source input point, the concentration levels from the different marine areas may be at the same order of magnitude. Even in the United States, a lot of the scientists declared that the contamination of PBDEs was the most serious one [38, 39]. It is said that nearly half of the total amounts of PBDEs of the world were used in America in 2001. Taking look at the research carried in San Francisco Bay sediments, the median values of ∑_8_PBDEs in each sample area (Suisun Bay, San Pablo Bay, San Pablo Bay, and South Bay) are about 2–6 ng/g. The median value of 22 samples representing the deep-water area of Strait of Georgia, ∑_8_PBDEs, is below 1 ng/g [40]. It is still comparable to the European samples (shown in Table 1), which seem to be the less polluted regions in the world according to the previous comments. In China, recently, not only the South China Sea but also the northern or eastern part of China Sea, such as BO Sea or East China Sea, has been investigated. Data indicate that, in the samples collected from offshore sediment of northern South China Sea, ∑_8_PBDEs is 0.93 ng/g [41] and from East China Sea it is below 1 ng/g [2], and all of median values of each published paper concerning northern part of China, for example, Laizhou Bay, Bo Sea, do not exceed 5 ng/g [42–44]. In terms of Korea, in surface sediments sampled from 8 less industrial activity or Shellfish farming or farming areas (Uljin, Ganggu, Kaduk Island, Wonmunnpo, Kohyonsong Bay, Gamak Bay, Sacheon Bay, Suncheon Bay, and Garorim Bay) the median value of the published ∑_14_PBDEs [45] is 1.3 ng/g. In Tokyo Bay, PBDE concentrations decreased from north (near Tokyo municipal areas) to south. Separating the impacted sites, ∑_8_PBDEs is 2.1 ng/g [33].

It seems that as long as the investigation sites located at a certain distance from the source pollution point, the PBDE concentrations would drop a lot, and finally from region to region they would not vary a lot. However, the pollution status from different countries is still not the same. It is hard to conduct a global monitoring program to precisely assess the pollution situation, and thus we take a look at the pollution sites; the values from diverse regions are quite different, some of the highest reported BDE-209 concentrations in sediments are 7340 ng/g in the Dongjiang River near a heavily industrialized area in southern China [41], and concentrations of BDE-209 in the Osaka Bay are (7.8–350 ng/dry wt) [46]. Conversely, in sediment at four areas in Pialassa Baiona coastal lagoon, spatial distribution of contaminants is affected by the location of anthropogenic inputs, the median of the sum of PBDEs is less than 6 ng/g [47], 17 surface sediments (top 2 cm) were sampled off the coast of Vancouver Island, British Columbia, Canada, and in the vicinity of the Capital Regional District's Clover Point municipal wastewater outfall, 2006, ∑_8_PBDEs is 0.75 ng/g [48]; Furthermore, in sample from east of Newcastle, Australian, described as Industry/urban area, the PBDEs still cannot be detected [49].

High levels of PBDEs were detected in sediments from river estuaries but drop quickly along with the distance from seashore. That is because, comparing with polluted river and some of the waste water receiving lake, owning to the distant from the input sources, marine sediment can receive much less pollutants. Besides, one of the major routes for pollutants going into ocean is riverine input. The high affinity of PBDEs to particulate and precipitate into the sediment may result in sedimentation and burial of PBDEs to the river sediment and significantly reduce the amount of transportable PBDEs to marine system [50].

### 3.2. Biota

**Figure 1:**
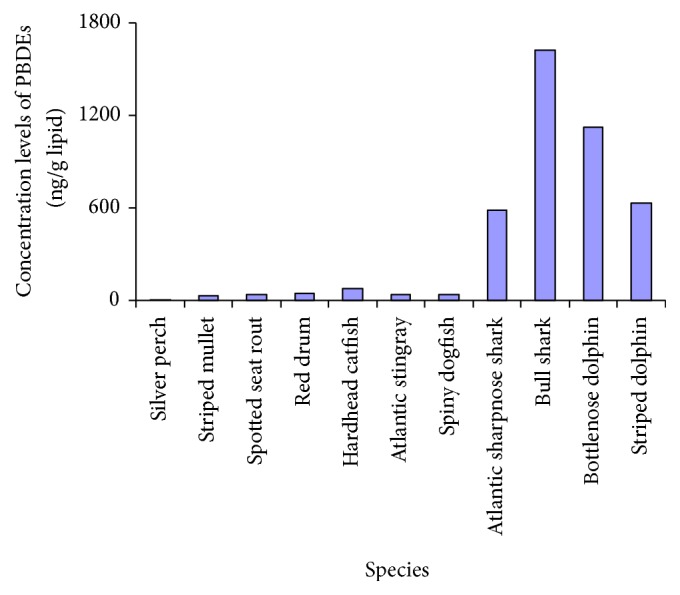
Mean value of lipid-normalized concentrations of ∑_8_PBDEs in different species from Florida coastal waters [52].

When comparing the data of sorts of biological samples, it is not wise to draw any conclusion just by looking at tissue levels without regard for the species variation. Concentrations of PBDEs in food webs from the Baltic Sea and the northern Atlantic Sea indicated that the detected ∑_8_PBDEs had a great difference between perch (17.13 ng/g lipid weight) and pike (111.94 ng/g lipid weight) [51]. According to published reports, PBDE levels in marine mammals may be generally one or more orders of magnitude higher than those in the invertebrates and fish collected from the corresponding sampling sites [52], as shown in Figure 1. Hence, in order to make a comparable data description, the discussion is limited to bivalves, which are the most commonly used bioindicator species [53, 54]. The median of lipid-normalized ∑_8_PBDEs levels in mussels collected from 25 coastal locations in Korea was detected at concentration of 142.74 ng/g lipid weight [55] (original data quoted based on the wet weight, recalculated by present authors). PBDE concentrations measured in these samples, excluding BDE-209, were lower than those reported from other countries, whereas BDE-209 concentrations were comparable to or higher than those reported from other countries. In their study, the predominant BDE congener in bivalves was Deca-BDE, which accounted for >60% of the total PBDEs [55]. Green-lipped mussels were used to investigate the concentrations of PBDEs in Hong Kong's marine environment. ∑_8_PBDEs ranged from 909.1 to 5545.4 ng/g lipid weight of mussel tissue, with a median high up to 2734.5 ng/g lipid weight [56]. Original data are quoted based on the dry weight, recalculated by present author using the lipid content, 1.1% derived from the data published by Phillips [53]. It was 2-3 orders of magnitude higher than that observed in France which was only 8.75 ng/g lipid weight in the mussels (in the absence of BDE-209). According to early study, mussel was collected from New Bedford Harbor, MA, USA, every alternate year from 1991 to 2005. The sum of the existing BDE congener (28, 47, 99, and 100, BDE-209 is not the target analysis conger) ∑PBDE concentrations in mussel tissues was in the ranges of 64 to 241 (mean, 135), 128 to 681 (mean, 295), and 128 to 364 (mean, 256) ng/g lipid weight at reference site, upper harbor, and lower harbor, respectively [37].

## 4. Controlling Factors

### 4.1. Sediment

The distribution patterns of PBDEs in sediments are complex and varied from area to area, as the distributions are controlled by several potential factors, including the source composition, environmental degradation, and sedimentary environment. Grain size and organic matter content are the main characteristics of sediments and the principal factors that control the sorption of hydrophobic organic contaminants [57–59].

PBDEs can be readily adsorbed on the particulate matters due to their high hydrophobicities [60]. Grain size can reflect the hydromechanic nature of deposition; it is very important to differentiate the sedimentary environment. It appears that the distribution of PBDEs pattern resembles to that of grain size as described in Jiaozhou Bay [61]. The covariation phenomenon was also demonstrated in Moon and his colleagues' work; grain size was described as factors that control the sorption of hydrophobic organic contaminants [55]. It is still hold true for the study in East China Sea. It presented a broad similar pattern between low-percentage sandy sediments and the concentrations of PBDEs [62], while the rule is not suitable for the South China Sea [41].

Because of their hydrophobicity, organic pollutants are frequently adsorbed to particles with high organic carbon contents in the environment. Some studies have reported that total organic carbon (TOC) is a principal factor that determines the adsorption of organic compounds, such as organic chlorine pesticides (OCPs) [63], polybrominated biphenyls (PCBs) [64], and chlorinated paraffin (CP) [65] in sediments from some regions. Several researchers also found a positive correlation between the distribution of PBDEs and organic carbon in sediment of a river estuary [4, 43]. However, poor correlations have also been observed between TOC and PBDEs in other areas [2, 41, 62, 66, 67].

The inconsistent distribution patterns may come from the following reasons. They may be due to the fact that researchers found that sediment organic matter differs in chemical composition and function among different size fractions. The coarse-size fractions may contain considerable inputs of coal and black carbon particles, leading to high OC concentrations. On the other hand, the higher OC concentrations of the finer-size fractions are often due to their large specific surface area and their high sorption for natural organic matter [58].

The congener-specific partitioning of halogenated contaminants onto sediments of differing sizes may be another potential reason. Generally, because of the greater organic content and surface area of smaller sediment fractions (i.e., colloidal and particulate organic carbon, organic detritus, silts, and clays), heavier congeners have a greater affinity for these sediments than smaller congeners based on equilibrium partitioning theory [68]. Rayner and his colleagues came up with a hypothesis that congener-specific partitioning occurs among sediment grains of different sizes and that the smaller grains are enriched in the contribution of higher brominated congeners such as BDEs-99 and BDEs-100 (only lightly brominated congeners from mono- through hexabrominated were discussed) [68]. Other studies have shown that heavier PBDE congeners are the most correlated ones with sediment grain size thus emphasizing the strong particle affinity of these compounds [69].

Besides, the possible reasons why the correlation between PBDEs and grain size or TOC is week may also be influenced by the intensive land-based inputs [70, 71], disturbed by occasional point source inputs [66], and degradation and reapportionment of PBDEs in water column during resuspension and long-range transport processes [60], or it can be concluded that it resulted from the combined effect of water dynamic, transport, mixing, and depositional mechanisms associated with PBDEs [41].

### 4.2. Biota

PBDE levels in biota can be influenced by many factors; besides environmental exposure, the deviations in species, sex, age, exposure duration, temperature, and latitude are all potential factors influencing bioaccumulation of PBDEs in marine organisms.

Apart from the PBDE concentrations in environmental medium, species distinction may be the dominated factor of all. Nine species of marine fish, including teleost fish, sharks, and stingrays, and two species of marine mammals (dolphins) collected from Florida coastal waters were analyzed for PBDEs. As shown in Figure 1, mean concentrations of PBDEs in different species fluctuated greatly from each other. The ∑_8_PBDEs levels measured in muscle tissues of teleost fish ranged from 6.5 ng/g lipid weight (in silver perch) to 77.4 ng/g lipid weight (in hardhead catfish). The levels may be about more than 10-fold higher in muscle of sharks ranging from 37.1 ng/g lipid weight (in spiny dogfish) to 1623.2 ng/g lipid weight (in bull sharks). In the blubber of marine mammals, high on the food chain such as bottlenose dolphins and striped dolphins, even the mean concentrations (1120.8 and 632.7 ng/g lipid weight, resp.) might be comparable to the maximum value in bull sharks. Concentrations of PBDEs in dolphins and sharks were 1-2 orders of magnitude greater than those in lower trophic-level fish species [52]. The same conclusion can be drawn by investigating the levels of individual PBDE congeners in various species from North Sea, such as invertebrate species (whelk, starfish, and hermit crab), the gadoid fish species (whiting and cod), and the marine mammal species (harbor seal and harbor porpoise). In the invertebrates, the ∑_8_PBDEs was found only to be ranging from 23.2 to 57.7 ng/g lipid weight. However, the ∑PBDEs levels in harbor porpoise liver and blubber were up to 1762 and 1555 ng/g lipid weight, respectively, which were generally one or more orders of magnitude higher than in the invertebrates [72].

The results obtained showed a positive correlation between trophic-level and PBDE concentrations [51, 72], which clearly points toward biomagnification potential of these chemicals [30]. Previous studies pointed out that the major biomagnification step in the food chain occurs from fish to marine mammals [72]. However, no clear trends could be discovered to suggest any biomagnification from benthic organisms to fish [73]. It can be partially interpreted by the differences in feeding behavior, nutritional status [74], and metabolic capacity. In addition, some researchers stated that body size would play an important role in the biomagnification processes. The size among the lower trophic-level species, such as fish and invertebrates, did not change a lot, whereas it could change a lot between the marine mammals and fish. The ratio of the total surface area of an animal to its size got smaller when size increased. The surface area of the gill membrane to total animal volume also had influence. Both factors resulted in enhanced partition of hydrophobic chemicals between the water and organisms due to body sizes of the organisms and lower elimination rates in larger animals [72, 75].

Even the same species may have different body burden levels of PBDEs. Previous studies had shown lower PBDE levels to occur in adult female marine mammals than in males, reanalysis of the data published by [76]; the median of adult female harbor seal (709.25 ng/g lipid) is lower than the male (1098.4 ng/g lipid). The reason was that they can dispose their contaminants during spawning by incorporating large amounts of fat and persistent pollutants in the roe [29, 73], and as a consequence pollutant transferred to their offspring during gestation and lactation [74].

The sampling time and metabolic capacity can influence the accumulation processes as well. The organisms suffering from the long-term exposure to the PBDEs may have higher body burden levels than that collected immediately after exposure, for the accumulation processes. Besides, the metabolic capacity played an important role in the processes, since some of the congeners may be metabolized and expelled as dejection.

## 5. Congener Distributions in Matrixes

**Figure 2:**
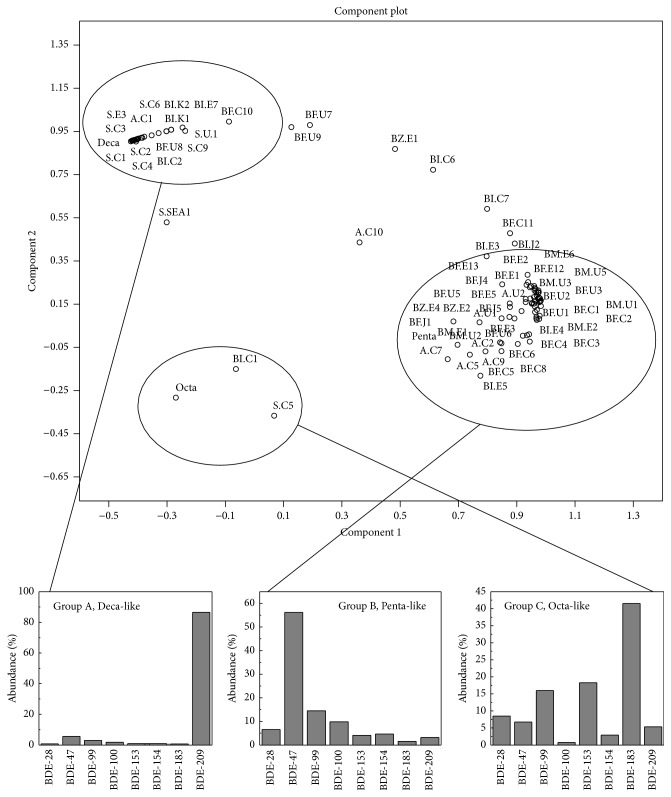
Plot of first two factors of Principal Component Analysis of PBDE congener patterns for matrixes (sediments, biotic species, and atmosphere) from Asia, America, and Europe.

The congener distribution patterns for the commercial mixtures (Penta-, Octa-, and Decamixtures) and for the samples of sorts of matrixes from Asia, Europe, and America are analyzed by Principal Component Analysis based on the proportion of eight individual PBDE congeners relative to the ∑_8_PBDEs concentrations. The samples were selected representing or associating with marine environment, such as sediments from estuary, coast, or open sea and biotic species (invertebrates, fish, or marine mammals) along with airborne PBDEs, which is one of the most important paths by which PBDEs transport into the ocean. The Principal Component Analysis plot revealed compositional similarity and differences both between and within environmental matrixes and commercial mixtures. The component plot of component 1 versus component 2 is shown in Figure 2. The first two principal components explained 88.95% of the total variability of the data set. As shown in Figure 2, the data points nominated as “∗.#*n*” indicated “∗” matrixes, “#” region, and “*n*” sample numbers. The abbreviations indicated the character of these samples: for matrixes, S is sediments; BI is invertebrates; BF is fish; BM is marine mammals; A is atmosphere; and for region, C is China; K is Korea; J is Japan; U is United States; E is Europe; SEA is Southeast Asia (detailed data description is listed in Table S1 in Supplementary Material available online at http://dx.doi.org/10.1155/2016/1317232). There is clear segregation of three-group cluster around the commercial mixtures. Group A has a similar compositional pattern to that of the Decamixtures, with relative predominant congener of 209. In this group, most of the data represent sediment samples, airborne particles, and some biological samples. Group B includes most of the biological samples which are similar to the Pentamixtures (Tetra- and Penta-BDEs are prevalent congeners) on the composition. The congener patterns will be discussed in detail in later section in this paper with the sequence of sample matrixes.

It is interesting to notice that some of the samples from China have a similar compositional pattern to that of the Octamixtures, as shown in Figure 2, uniquely gathering in group C.

The samples collected from China, unlike those collected from other countries, are similar to the Octamixtures and have a relative high proportion of BDE-183 to the total load of ∑_8_PBDEs (not only the sediment sample, some of the human sample also showed the same distribute pattern; see our previous publication [77]). This unexpected phenomenon can be interpreted by the fact that China is the major port of electronic-waste (e-waste). BDE-183 can be an indicator congener of Octamixtures, which are primary used in the resin or polymer applied to electronic industry [78, 79]. The use of Octamixtures has been banned in all applications in the European Union Market since August 2004 (Brominated Science and Environmental Forum web site), and the Great Lake Chemical Corporation (USA) had agreed to phase out the production of the chemicals by the end of 2004 [80]. However, the electronic applications produced in America and Europe years before 2004, incorporated with these toxic chemicals, may come to the end of their “life span” and be discarded as e-waste years after ban-time. Instead of being recycled locally, these e-wastes carrying the toxic Octamixtures have been exported to developing countries, such as China. Therefore, some of the samples collected from China may have high proportions of BDE-183, similar to Octamixtures in congener patterns, uniquely gathering in the group C.

### 5.1. Sediment

As shown in Figure 2, on the top left corner of the figure, most of the sediment samples cluster together (group A), which are similar in compositional pattern to the Decamixtures. That is because the moderately and heavily substituted BDE congeners predominate over the less brominated congeners in the sediments, especially BDE-209, which is the major congener detected in the two Decamixtures (Saytex 102E and Bromkal 82-0DE) accounting for 96.8% and 91.6% for the total load, respectively [27], in the meantime, dominating the congener patterns in the sediments representing over 85% of the ∑_8_PBDEs in each study, except the samples from Hong Kong [56]. The levels of BDE-209 are 1–3 orders of magnitude higher than the sum of the concentrations of Tri- to Hepta-BDEs in some studies. There are reasons accounting for the abundance of BDE-209 in the profiles. PBDEs were reported to be introduced into marine system through wet and dry deposition [81]. Studies indicated that the percentage of contaminant adsorbed to particles in both air and wet deposition increased with bromination [81]. Studies showed that compositions of PBDEs at urban and suburban sites were similar, in which BDE-209 was the dominated congener [82], with an annual average contribution to ∑PBDEs of 64 ± 23% in an across-China study [83] or 40–99% in south China. In these samples BDE-209 was the predominant one over the 11 congeners investigated, accounting for 40–99% for the total PBDE concentrations in the atmosphere samples, except for some irregular site as stated [28]. Along with the determination from deposition, the physical-chemical properties of the individual congeners play an important role as well. According to the octanol-water partition coefficient, it is expected that the heavier brominated congener (such as BDE-209) has high affinity to particulate and precipitate into the sediment [7]. Furthermore, BDE-47 and BDE-99 and some other lighter congeners are easier to be taken up by organism while not being restrict into the sediment. All of the reasons above may lead to the predomination of BDE-209 in the congener patterns in sediment.

### 5.2. Biota

PBDE congener patterns of most biota from all over the world seem to resemble to each other, irrespective of levels, fish species, and sampling sites. Most of the data points cluster together with the Pentamixtures. BDE-47 and BDE-99 are the main components of the Pentamixtures, including DE-71 (which has a composition of 38.2% and 48.6%) and Bromkal 70-5DE (which has a composition of 42.8% and 44.8%) [27]. The percentage of BDE-209, which is the main component of Decamixtures, is much lower than that of BDE-47 and BDE-99 in biological samples. The general order of decreasing contribution to the total load is BDE-47 > BDE-99, BDE-100 > BDE-153, BDE-154. BDE-47, BDE-99, and BDE-100 make up about 80% on average of the ∑_8_PBDEs. It is clear that in biological samples the congeners are dominated by the light brominated ones and are obviously different from those in sediment and airborne particle samples, which are dominated by BDE-209 [84]. The distinction comes from the different bioavailability between those congeners. Bioaccumulation of PBDEs with six or more bromine atoms seems to be correlated negatively with the degree of bromination [72]. The reason may be that the relatively high molecular weight (644–959 Dalton) or molecular size leads to inefficient dietary uptake [29].

As shown in Figure 2, not all biological samples cluster around the Pentamixtures. The congener patterns of some biotic species are much more similar to those of Decamixtures than others, because the samples contain more BDE-209 than other species. BDE-209 has been considered to be nonbioavailable, because its large molecular size may impede its passage across tissue membranes in biota [29]. However, some studies have demonstrated that organisms were able to take up BDE-209. In laboratory, experiments were carried out to confirm that the BDE-209 could be taken up by carp, and uptake of BDE-209 was estimated to be 3.2% [85]. But the accumulation efficiency is extremely low. It has been proved that BDE-209 can be metabolized by juvenile carp, since no net accumulation of BDE-209 was observed throughout the experiment despite an exposure concentration of 940 ng/day/fish [85]. The same metabolic capability can be observed in rainbow trout. Dietary uptake and effects of BDE-209 were studied in rainbow trout. Fish were fed Deca-BDE for 49 days and then on control diet for 71 days to study depuration. After depuration, BDE-209 concentrations declined. Generally, as a result of biotransformation processes, BDE-209 has a short half-life in organisms [72, 73]. No matter whether the BDE-209 can be taken up by organisms or not, the rapid metabolism and elimination of this congener may sequentially result in its absence in biota as well. To our knowledge, no integrated study has figured out the relationship between metabolic capacity and taxonomic group. The absence and presence of BDE-209 can be attributed to the metabolic differences between species and the depuration time somehow. The irregular points (BZ.E1, BI.E7, BF.U7, BF.U8, and BF.U9) include the zooplanktons, roach [29], and sharks (spiny dogfish, Atlantic sharpnose shark, and bull shark) [52]. It seems that these species, compared with other marine organisms, can accumulate the BDE-209 effectively and have a relative long residue time.

Besides the reasons mentioned above, another significant reason should be taken to account for the elevated proportion of BDE-209. In Figure 2, the data point (BI.K1) of bivalves collected from Korean coastal belongs to group A, with a high percentage of BDE-209 of the total load. This is due to the approach of the sample preparation. In their study, the mussels analyzed were not depurated, and the whole soft tissues were pooled and homogenized [55]; therefore, the presence of particles in the gut may contribute to the high BDE-209 concentration measured. Filter-feeding mussels ingested the contaminants adsorbed on small particles [55]. Booij et al. [86] suggested that BDE-209 levels measured in mussels were dominated by the concentrations found in ingested particles in the gut. This also can explain the data point BI.C2, representing for the mussel from China.

**Figure 3:**
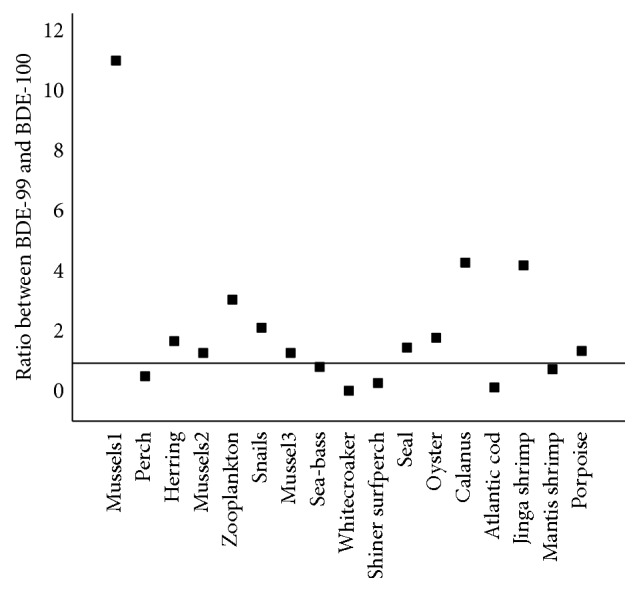
The ratio between BDE-99 and BDE-100 for different species. The lower trophic-level species, like mussels and shrimp (lake of metabolic capability), often turns to be greater than 1, while for those higher species, like fish or marine mammals (elimination of BDE-99), the ratio may be below 1. It is worthy to mention that some species feed mainly on the crustaceans and zooplankton seems to have a higher ratio value, as mentioned in the manuscript (data are collected from references [29, 44, 51, 55, 56, 72, 76, 87, 99–101]).

In the present study, different ratios between BDE-99 and BDE-100 were calculated in different species (as shown in Figure 3). The statement made earlier by Christensen et al. has shown that the mean ratio between BDE-99 and BDE-100 in marine environment locations which were not excessively polluted was equal to 30 : 70. Actually, however, according to our analytical data, it is evident that the ratio is much like the species-depended. The ratio varied in species even though the samples were collected from the same region. In addition to the exposure ambient, molecular size, and bioavailability, the metabolic differences between the species would be an important reason accounting for the variance. Voorspoels et al. have observed that the ratio found for shrimp (20 : 80) was very similar to the ratio for the Bromkal mixtures and virtually identical to the ratio found in Western Scheldt Estuary sediments [73]. This result was consistent with the measured ratio in shrimp and the sediments of the Pearl River estuary [87]. Shrimp simply reflected the Bromkal and sediment constitution patterns for these congeners, suggesting that these compounds were readily bioavailable, but shrimp clearly lacked metabolic capability [87]. Hites reported that the selective environmental elimination of BDE-99 had been observed in some biota [88]. The marine organisms seemed to increase their metabolic capabilities when they climb the evolutionary ladder [73]. The ratio between BDE-99 and BDE-100 for the lower trophic-level species, like mussels, snail, shrimp, and* Calanus*, and so forth (lake of metabolic capability), often turns to be greater than 1, while for those higher species, like fish or marine mammals (elimination of BDE-99), the ratio may be below 1. It is worthy to mention that some species feed mainly on the crustaceans and zooplankton seems to have a higher ratio value, such as herring [51, 52, 72], striped mullet [52], or stingray [52] (as shown in Figure 3).

### 5.3. Atmosphere

As mentioned above, atmosphere transportation is one of the major routes for PBDEs to transport, redistribute, and finally deposit into marine system. To estimate the load of PBDEs to marine system, the atmospheric compositional pattern should be figured out to quantify and predict their environmental fate and transport.

As shown in Figure 2, some of the atmospheric samples (A.C1, A.C3) belong to group A, which might be similar to the Decamixtures in composition. On the contrary, the samples (A.C2, A.U1, and A.U2) belong to group B which might have a similar compositional pattern to that of the Pentamixtures. That is due to the atmospheric PBDE partitions between the vapor and particulate phases. On the basis of the KOA values [26], it can be predicted that the lighter congeners (e.g., BDE-47, BDE-99, and BDE-100) will exist almost entirely in the gas phase, whereas the heavier brominated congeners (BDE-153, BDE-154, and BDE-183) will be predominantly associated with particles [26]. BDE-209, according to its physical-chemical properties (e.g., a log subcooled liquid vapor pressure, *V*_*p*_ of −8.68, and an octanol-water partition coefficient, *K*_ow_, of 9.97), is expected to be partitioned entirely to the particle phase in both air and rain [81].

Taking the gas-particle partitioning of PBDEs into consideration, the compositional pattern for particulate phase is similar to that of sediments (group A), in which the relative abundance of BDE-209 is extremely high as well. This can be served as a potent evidence for the fact that wet or dry deposition is one of the major sources of the PBDEs in marine sediments, whereas the gas phase has a similar congener pattern to that of biological samples (group B), and the lighter congeners contribute more than heavier ones to the total load.

Atmosphere samples were collected from four sites in the city of Guangzhou, a typical urban center in south China to determine the gas-particle partitioning of 11 PBDE congeners. The average relative abundances of PBDEs have shown that the Tetra- to Hepta-BDEs were present in both the gas and particulate phases. The Tri-BDE (BDE-28) was present almost exclusively in the gas phase (96–98%) [28]; therefore, the data point of gas phase sample (A.C2) belonged to group B, whereas the Deca-BDE (BDE-209) was found only in the particulate phase [28], resulting in clustering together with group A. In some studies the atmosphere samples contain both the particle and gas phases. It becomes difficult to predict whether it will have a Deca-like profile or Pentaprofile, since it depends on which phase will be the dominant one.

Along with the gaseous or particulate phases of samples, the sample location may significantly influence the composition profile of atmosphere sample. In samples collected from urban or suburban sites in China, the dominating conger is BDE-209, and background/rural air sites, however, are BDE-47 followed by BDE-99. These differences can be explained as BDE-209 is easy to bound to particles and hence less subject to migrate with air mass movement to the background/rural sites in comparison to other less brominated PBDEs [83]. In both urban and background/rural sites, the ratio for BDE-209 to the total PBDE in Chinese ambient air was greater than that in air of the United States [89], while the ratios for BDE-47 and BDE-99 in Chinese air were smaller than those in the US air, which is possibly due to the larger usage of Pentamixtures in the United States [89] and Decamixtures in China [83].

## 6. Recommendations and Data Gaps

Marine system plays an important role in the environment [24]. However, studies on PBDEs in marine system are patchy and fragmented. Most of the sampling sites have been conducted in the bay or near coastal area, which could be strongly affected by municipal and industrial wastewaters containing high levels of anthropogenic pollutants. Some of sample locations even are situated near point sources such as harbors or wastewater treatment plants, where concentrations in various environmental matrixes are predicatively to be relatively high [40]. For a complete understanding of the distribution of PBDEs in marine system, the investigation should be expanded geographically. Such background locations and open sea area should also be included.

The congeners determined varied from study to study, which has been an impediment for comparing the conclusion across various studies. The different polluted level may come from how many and which congeners are determined, rather than actual polluted status. BDE-209 is of special importance because it is suspected to be the predominant congener in the sediments and it may account for over 85% in the samples. Owing to the unmeasurement of BDE-209, the pollution level would be underestimated. The US Environmental Protection Agency has made a list of 16 “priority pollutant polycyclic aromatic hydrocarbons (PAHs)” that are indicative for monitoring PAH contaminations. There is an urgent need to make a list to guide the researchers in determining congeners to whom priority investigation are warranted.

To quantify and predict their environmental behaviors of PBDEs in the marine system, there is a clear need for more systematic environmental monitoring to understand how and where these chemicals are being released into the environment and what is happening to them once they enter the environment. But the researches focused their attention ex parte on the sediments and biota [7]. Extra emphasis should be placed upon the water column, suspended particles, microlayer, and pore-water and properties in environment such as redox conditions and salinity or some other geoenvironmental parameter worth for future studies, to understand what are the fluxes of these chemicals into marine system, what is happening to them once they enter the environment, and what fate and transport processes that are involved in their environmental movement.

Some of previous studies paid attention to the debromination process of these chemicals in the lab; modeled or field study of PBDEs in real situation is urgently needed. It is a major knowledge gap to understand what debromination process these chemicals undergo in the practical situation. Can these chemicals be hydrolyzed, photolyzed, reductively decomposed in the water, particle, and sediment, or metabolized by marine organisms? And if yes, what are the resultant products? Do these degradation products turn to be more deleterious or harmless to the organisms? And is there any appropriate way to degrade or assimilate the increasing PBDEs in marine system? There are still a lot of questions waiting to be resolved.

The Penta-BDE and Octa-BDE commercial mixtures have been banned in the European Union (EU) in 2004 and were included in the Stockholm Convention of Persistent Organic Pollutants (POPs). In addition, Deca-BDE has also been proposed for listing under the convention [15]. Despite the ending of the production and use, large quantities of articles containing PBDEs are still in use as well as in the recycling and end-of-life flows [90]. Therefore, some trend study, such as taking sediment core as information carrier or using material flows approach, should be done to understand or predict the existing and potential risk.

## Supplementary Material

In this paper we provided a meta-analysis results of a bunch of literatures. The calculating result may be different from the data present in original paper. It is because instead directly using published data, we make a whole though reanalysis of the existing data to meet our report theme, such as congener and sample site selection. In order to provide a better comparison of each work, we choose 8 congeners (BDE-28, BDE-47, BDE-99, BDE-100, BDE-153, BDE-154, BDE-183, BDE-209) those frequently reported and play important roles to the total load. According to our report theme, some of the sample sites are eliminated, including the ones located at the inner river, point source input. Based on the selection criteria mentioned above, in this supplementary material, we reanalysis the original data and calculate the ratio of each congener to the total load (Σ_8_ PBDEs) to represent the difference of the composition of 8 selected congeners in different environmental matrixes from different regions.
The detailed information of the congener composition of each environmental matrixes is discussed in the text. The data points nominated as “∗.#*n*” indicated “∗” matrixes, “#” region, and “*n*” sample numbers. The abbreviations indicated the character of these samples, for matrixes, S is sediments; BI is invertebrates; BF is fish; BM is marine mammals; A is atmosphere; and for region, C is China; K is Korea; U is United States; E is Europe; SEA is Southeast Asia.

## Figures and Tables

**Table 1:** Summary of worldwide marine sediment PBDE levels (sum of 8 selected PBDE congeners: BDE-28, BDE-47, BDE-99, BDE-100, BDE-153, BDE-154, BDE-183, and BDE-209).

Region	Sample site	∑_8_PBDEs ng/g	Data description	Reference
*Asian*				

China	Nine sediment samples were collected around the coast of Macao, which was known asdepositional zone, 2002.9	26.85	The median of the 9 data sets, the missing values are treated and replaced with 0	[41]
	14 offshore sediment samples were collected in the northern SCS, 2002.7	0.93	The median of the 14 data sets, the missing values are treated and replaced with 0	[41]
	26 marine samples were collected from Laizhou Bay area, North China, 2009. 9-10	4.83	Given median BDE-209 plus given median ∑_7_PBDE	[42]
	Six sediment samples were collected from offshore sediment sample near Daliao River in spring (May) 2007	0.20	take sp7, sp8, and sp10–sp13 to represent the open sea sample, the median of the 6 data sets	[43]
	Six sediment samples were collected from offshore sediment sample near Daliao River in summer (August) 2007	0.43	take su7, su9-su10, and su12–su14 to represent the open sea sample, the median of 6 data sets	[43]
	Five sediment samples were collected from Daliao River open sea in autumn (November) 2007	0.34	Take au7, au10–au12, and au14 to represent the open sea sample, the median of 5 data sets	[43]
	16 surface sediments were sampled from Bo Sea in August 2006	2.96	Sum of the published median value of each congener	[44]
	24 sediment cores (from depths of approximately 30 cm below the seawater) were collected at monitoring stations in the East China Sea during October and November, 2013	0.46	Sum of the published median value of each congener. The missing values are treated and replaced with 0	[2]
	Samples of surface sediments (up to approximately 10 cm depth) were removed close to the shoreline from 13 locations in Hong Kong coastal area	7.56	Mean value of the 26 data sets (each sample site has duplication in the published data)	[56]

Korea	Surface sediments (at a depth of 0–4 cm) were sampled at the industrialized bays Ulsan Bay; sediment samples were collected systematically from the inner (including rivers or streams) to outer parts of the bay from February 2003 to March 2004	31.17	Sum of published median value of each congener	[91]
	Surface sediments (at a depth of 0–4 cm) were sampled at the industrialized bays, Busan Bay, from February 2003 to March 2004	59.76		[91]
	Surface sediment (at a depth of 0–4 cm) was sampled at the industrialized bays, Jinhae Bay, from February 2003 to March 2004	9.53		[91]
	Surface sediments were sampled from Busan Bay in the coastal waters of Korea in 2005	77.50	Median of the 4 data, ∑_8_PBDEs is not available; take the published ∑_14_PBDEs as substitution	[45]
	Surface sediments were sampled from 8 industrial complex bays (Yeongil Bay, Ulsan Bay, Onsan Bay, Masan Bay, Haengnam Bay, Gwangyang Bay, Asan Bay, and Kyeongi Bay) in the coastal waters of Korea in 2005	12	Median of 10 data sets, each bay has one sample, except Ulsan and Onsan which have two, ∑_8_PBDEs is not available, and take the published ∑_14_PBDEs as substitution	[45]
	Surface sediments were sampled from 8 less industrial activity or shellfish farming or farming areas (Uljin, Ganggu, Kaduk Island, Wonmunnpo, Kohyonsong Bay, Gamak Bay, Sacheon Bay, Suncheon Bay, and Garorim Bay) in the coastal waters of Korea in 2005	1.30	Median of 9 data sets, each bay has one sample, ∑_8_PBDEs is not available, and take the published ∑_14_PBDEs as substitution	[45]
	Surface sediments were sampled at 25 locations from the Korean coast from February to June 2004	4.39	Sum of the median of each congener	[55]

India	10 surface sediments were collected from the open Indian Ocean at depths below 4000 m in 2011	0.15	The median of the 10 data sets	[92]

Japan	Three sediment core samples were taken from the northern part of Tokyo bay; surface sediments were taken to calculate the median value	46	The ∑_8_PBDEs is not available; take the median of the BDE-209 of 3 surface sediments as substitution	[33]
	Six surface sediment samples were taken from the southern part of Tokyo bay	2.1	The ∑_8_PBDEs is not available; take the median of the BDE-209 of 6 surface sediments as substitution	[33]

Tropical Asian region	Sediment core samples from Philippines, in Southeast Asia, were collected in December 2009; surface sediment were taken to calculate ∑_8_	2.36	Congener concentration below detected limit replaced it with 0	[78]
	Sediment core samples from the upper Gulf of Thailand were taken in June 2004; surface sediments were taken to calculate ∑_8_	1.24		[78]

*Europe*				

Spain	Sediment samples were collected from several hot spots on the Spanish coast, such as the harbors of Almeria and Tarragona and mouths of Besós and Llobregat rivers in Barcelona	4.55	The median of the 12 data sets (B1 has been excluded, for being collected from River Llobregat mouth), if fewer than 50% (half of the detected level), the missing values are treated with the CR ratio; if not, replaced with 0	[93]

Denmark	Surface sediments were collected at 10 different locations in Danish marine territory, BDE-209 has been measured in 6 of the 10 samples; here we calculated the 6 ones	2.42	Median of the published data sets, missing values are treated with the CR ratio	[94]

Holland	Sampling sites were chosen so that different parts of the Dutch sector of the North Sea continental shelf were covered, in spring of 2000	8.68	Median of the 8 published data sets (samples 1 and 3 have been excluded, for being collected from Scheldt estuary outflow and Rhine-Meuse estuary and Rotterdam harbor); missing values are treated by the CR ratio	[95]

Belgium	Six location in Belgian North Sea (S1–S6) were sampled	2.16	Median of samples S1–S6, missing number treated as half of the detected limit	[96]

Norway	Five surface sediments were collected from Tromsøflaket, in the Barents Sea, in 2006	0.07	Median of 5 data sets (Rx), missing number treated as half of the detected limit	[97]

Italy	Sediment at 4 areas in Pialassa Baiona coastal lagoon, spatial distribution of contaminants is affected by the location of anthropogenic inputs	4.7	Target BDE congeners: BDE-3, BDE-7, BDE-15, BDE-17, BDE-28, BDE-47, BDE-49, BDE-66, BDE-71, BDE-85, BDE-99, BDE-100, BDE-119, BDE-139, BDE-153, BDE-154, BDE-183, BDE-196, BDE-197, BDE-201, BDE-203, BDE-204, BDE-206, BDE-207, and BDE-208; ∑PBDEs is 0.58 *μ*g/kg; we take the BDE-209 (4.7 *μ*g/kg) as substitution	[47]

*North America*				

Canada	Twenty-two deep-water sediment samples were collected from the Strait of Georgia between 2003 and 2007	0.64	Median of the 22 data sets, BDE-28, BDE-153, BDE-154, and BDE-183 are not available; we take the ∑_4_ as substitution	[40]
	Sediment samples (at a depth of 6 cm) were collected at Bazan Bay in the Strait of Georgia between 2003 and 2007	0.63	The published median value	[40]
	Sediment samples (at a depth of 4 cm) were collected at Blubber Bay in the Strait of Georgia between 2003 and 2007	0.08		[40]
	Sediment samples (at a depth of 4 cm) were collected at Burrard Inlet in the Strait of Georgia between 2003 and 2007	11.02		[40]
	Sediment samples (at a depth of 3 cm) were collected at Cowichan Bay in the Strait of Georgia between 2003 and 2007	0.33		[40]
	Sediment samples (at a depth of 13 cm) were collected at Descanso Bay in the Strait of Georgia between 2003 and 2007	0.32		[40]
	Sediment samples (at a depth of 29 cm) were collected at Deep Cove in the Strait of Georgia between 2003 and 2007	0.37		[40]
	Sediment samples (at a depth of 5 cm) were collected at Scuttle Bay in the Strait of Georgia between 2003 and 2007	0.18		[40]
	17 surface sediment (top 2 cm) were sampled off the coast of Vancouver Island, British Columbia, Canada, in the vicinity of the Capital Regional District's Clover Point municipal wastewater outfall, 2006	0.75	Median of the 17 data sets	[48]

US	Surface sediments (top 5 cm) were collected from the Suisun Bay in 2007	3.87	The published median value, for BDE-183 is not available, replace it with 0	[76]
	Surface sediments (top 5 cm) were collected from the San Pablo Bay in 2007	2.46	The published median value, for BDE-183 is not available, replace it with 0	[76]
	Three surface sediments (top 5 cm) were collected from the Central Bay in 2007	3.24	Median of the 3 data sets, for BDE-183 is not available, replace it with 0	[76]
	Two surface sediments (top 5 cm) were collected from the South Bay in 2007	3.15	Median of the 2 data sets, for BDE-183 is not available, replace it with 0	[76]
	Three surface sediments (top 5 cm) were collected from the Lower South Bay in 2007	5.14	Median of the 3 data sets, for BDE-183 is not available, replace it with 0	[76]

*Others*				

Australia	East of Newcastle described as industry/urban area	0	All the PBDEs congeners are under detective limit, except BDE-28 which the data did not show in paper	[49]

Chile	Three samples wear collected in Concepción Bay, Chile, in December 2009	1.98	Median of the 4 data sets, since the median or mean value is not given in the present article, the max concentrations of each congener are taken to calculate the sum of available PBDE congeners (BDE-47, BDE-99, BDE-100, BDE-154, BDE-183, and BDE-209)	[98]
	Four samples wear collected in San Vicente Bay, Chile, in December 2009	2.11		[98]
	Four samples wear collected in Coronel Bay, Chile, in December 2009	2.42		[98]

Colombia	Four samples were collected in West Coastal Line of Colombia in April 2010	0	Median of the 4 data sets, since the median or mean value is not given in the present article, the max concentrations of each congener are taken to calculate the sum of available PBDE congeners (BDE-47, BDE-99, BDE-100, BDE-154, BDE-183, and BDE-209)	[98]
