# Supplementary material for: Environmental Characteristics of Polybrominated Diphenyl Ethers in Marine System, with Emphasis on Marine Organisms and Sediments
Source: Biomed Res Int. 2016 Nov 24;2016:1317232. doi: 10.1155/2016/1317232 (PMC5143782; doi:10.1155/2016/1317232)
Supplement: Supplementary file 1 — In this paper we provided a meta-analysis results of a bunch of literatures. The calculating result may be different from the data present in original paper. It is because instead directly using published data, we make a whole though reanalysis of the existing data to meet our report theme, such as congener and sample site selection. In order to provide a better comparison of each work, we choose 8 congeners (BDE-28, BDE-47, BDE-99, BDE-100, BDE-153, BDE-154, BDE-183, BDE-209) those frequently reported and play important roles to the total load. According to our report theme, some of the sample sites are eliminated, including the ones located at the inner river, point source input. Based on the selection criteria mentioned above, in this supplementary material, we reanalysis the original data and calculate the ratio of each congener to the total load (Σ8 PBDEs) to represent the difference of the composition of 8 selected congeners in different environmental matrixes from different regions. The detailed information of the congener composition of each environmental matrixes is discussed in the text. The data points nominated as “∗.#n” indicated “∗” matrixes, “#” region, and “n” sample numbers. The abbreviations indicated the character of these samples, for matrixes, S is sediments; BI is invertebrates; BF is fish; BM is marine mammals; A is atmosphere; and for region, C is China; K is Korea; U is United States; E is Europe; SEA is Southeast Asia. [file 1317232.f1.doc]

**Supplementary Information**

Table S1 Description of data point in Figure 2

| Data point ID. | Matrix description | Ratio to Σ8 | | | | | | | | Ref. |
| --- | --- | --- | --- | --- | --- | --- | --- | --- | --- | --- |
|  |  | BDE-28 | BDE-47 | BDE-99 | BDE-100 | BDE-153 | BDE-154 | BDE-183 | BDE-209 |  |
| Penta | Commercial product DE-71 | 0.23 | 34.6 | 44.1 | 11.9 | 4.94 | 4.12 | 0.09 | 0.00^1^ | [1] |
| Octa | Commercial product DE-79 | 0.00 | 0.00 | 0.00 | 0.00 | 16.3 | 2.02 | 79.2 | 2.47 | [1] |
| Deca | Commercial product Saytex102E | 0.00 | 0.00 | 0.00 | 0.00 | 0.00 | 0.00 | 0.00 | 100 | [1] |
| S.C1 | River sediment, three major rivers of Pearl River Delta | 0.05 | 0.76 | 0.16 | 1.03 | 0.15 | 0.26 | 0.23 | 97.4 | [2] |
| S.C2 | Coastal sediment, coast of Macao | 0.07 | 1.35 | 0.61 | 7.58 | 1.56 | 2.30 | 1.01 | 85.5 | [2] |
| S.C3 | Open sea sediment, northern South China Sea | 0.63 | 3.77 | 0.63 | 4.40 | 0.00 | 0.00 | 0.00 | 90.6 | [2] |
| S.C4 | Estuarine sediment, along the Pearl River Delta | 0.42 | 2.81 | 0.36 | 2.60 | 0.31 | 0.57 | 0.36 | 92.6 | [2] |
| S.C5 | Marine sediment, close to the Hongkong shoreline | 10.2 | 10.2 | 26.4 | 0.38 | 21.3 | 4.27 | 23.9 | 3.28 | [3] |
| S.C6 | 16 surface sediments (0–5 cm), Bo Sea, North of China, in August 2006 | 1.69 | 10.81 | 6.08 | 1.35 | 1.01 | 0.68 | 1.01 | 77.36 | [4] |
| S.C7 | Surface sediments, East China Sea, October and November, 2013 | 4.59 | 13.32 | 5.68 | 0.00 | 0.00 | 0.00 | 0.00 | 76.42 | [5] |
| S.C8 | Sediment samples from Daliao River Estuary, north of China, 2007 | 0.86 | 1.28 | 0.83 | 0.14 | 0.67 | 0.37 | 0.55 | 95.72 | [6] |
| S.C9 | 16 surface sediments (0–5 cm), Bo Sea, North of China, in August 2006 | 1.69 | 10.81 | 6.08 | 1.35 | 1.01 | 0.68 | 1.01 | 77.36 | [4] |
| S.C10 | 12 Surface sediments, Xiamen offshore areas, southeast of China. 28=28/33 | 0.49 | 2.60 | 2.70 | 0.53 | 3.99 | 1.46 | 2.25 | 85.10 | [7] |
| S.C11 | Riverine sediments (n=36), Laizhou Bay, north of China | 1.51 | 1.40 | 1.12 | 0.11 | 0.17 | 0.17 | 0.17 | 95.34 | [8] |
| S.C12 | Marine sediments (n=26), Laizhou Bay, north of China | 1.23 | 1.23 | 1.23 | 0.41 | 2.26 | 0.62 | 0.62 | 92.40 | [8] |
| S.U.1 | Suspended sediments from the Detroit River. Single-point sediment were deployed from April to November in the Detroit River ranging from the mouth at the outflow to western Lake Erie to the head in southern Lake St. median value | 0.32 | 13.05 | 15.01 | 3.17 | 1.73 | 1.46 | 0.58 | 64.88 | [9] |
| S.SEA1 | Ten surface sediments were collected from the open Indian Ocean at depths below 4000 m in 2011. | 4.88 | 10.61 | 4.67 | 21.52 | 1.74 | 23.51 | 5.54 | 26.65 | [10] |
| S.SEA2 | Surface sediment from core sample from taken from the upper Gulf of Thailand. | 0 | 0.11 | 0.19 | 0.12 | 0.19 | 0.27 | 0 | 99.13 | [11] |
| BI.C1 | Green-lipped mussels, shell lengths between 60-120mm, close to the Hongkong shoreline | 15.3 | 9.99 | 21.5 | 1.89 | 17.2 | 2.46 | 21.4 | 10.3 | [3] |
| BF.C1 | Fish samples, samples were collected from six rivers and three estuaries in Taiwan | 3.01 | 62.7 | 7.59 | 8.78 | 11.6 | 4.58 | 1.67 | 0.00 | [12] |
| A.C1 | Gas phase of the Atmosphere samples, encompass a range of aerosol types and potential PBDE sources in Guangzhou City | 2.39 | 6.89 | 6.85 | 1.00 | 1.56 | 0.71 | 0.84 | 79.8 | [13] |
| A.C2 | Gas phase of the Atmosphere samples, encompass a range of aerosol types and potential PBDE sources in Guangzhou City | 15.1 | 40.4 | 33.9 | 5.37 | 2.57 | 2.35 | 0.36 | 0.00 | [13] |
| A.C3 | Particle phase of the Atmosphere samples, encompass a range of aerosol types and potential PBDE sources in Guangzhou City | 0.08 | 1.02 | 2.40 | 0.26 | 1.34 | 0.45 | 0.92 | 93.5 | [13] |
| A.U1 | Air samples, two rural locations, one at Sleeping Bear Dunes, on the northeast coast of Lake Michigan and one at Sturgeon Point, located about 50m from the shore of Lake Erie,1999 | - | 48.0 | 39.3 | 6.14 | 3.70 | 2.05 | - | 0.79 | [14] |
| A.U2 | Air sample, an urban sampling site on the south side of downtown Chicago, approximately 1.6 Km from the shore of Lake Michigan | - | 61.2 | 32.1 | 3.21 | 1.49 | 1.05 | - | 0.99 | [14] |
| A.C5 | The gaseous phase of atmosphere samples collected from urban area across China (11 cities) were collected from September 2008 to August 2009 | 34.29 | 38.72 | 12.33 | 3.52 | 5.89 | 3.18 | 2.07 | 0.00 | [15] |
| A.C6 | The particulate phase of atmosphere samples collected from urban area across China (11 cities) were collected from September 2008 to August 2009 | 0.85 | 2.86 | 5.42 | 0.69 | 3.71 | 1.21 | 2.39 | 82.88 | [15] |
| A.C7 | The gaseous phase of atmosphere samples collected from 75 km away from the downtown of Shanghai, China were collected from September 2008 to August 2009 | 40.28 | 38.63 | 10.75 | 4.53 | 1.70 | 1.84 | 2.27 | 0.00 | [15] |
| A.C8 | The particulate phase of atmosphere samples collected from 75 km away from the downtown of Shanghai China were collected from September 2008 to August 2009 | 1.24 | 5.72 | 5.38 | 1.62 | 2.54 | 2.23 | 10.64 | 70.63 | [15] |
| A.C9 | The gaseous phase of atmosphere samples collected from 3 background/rural sites of China were collected from September 2008 to August 2009 | 15.66 | 37.37 | 31.41 | 8.34 | 2.99 | 2.26 | 1.96 | 0.00 | [15] |
| A.C10 | The particulate phase of atmosphere samples collected from 3 background/rural sites of China were collected from September 2008 to August 2009 | 0.74 | 22.35 | 35.49 | 6.59 | 6.87 | 2.90 | 4.44 | 20.61 | [15] |
| BF.U1 | Silver perch muscle, n=6, Samples were collected from estuarine waters of the Indian River Lagoon and adjacent offshore coastal waters of Florida in 2004 | 4.61 | 55.29 | 26.1 | 6.14 | 3.07 | 3.07 | 1.54 | 0.17 | [16] |
| BF.U2 | Striped mullet muscle, n=6, Samples were collected from estuarine waters of the Indian River Lagoon and adjacent offshore coastal waters of Florida in 2004 | 3.71 | 72.3 | 8.44 | 5.07 | 3.71 | 6.75 | 0.00 | 0.04 | [16] |
| BF.U3 | Spotted seatrout muscle, n=7, Samples were collected from estuarine waters of the Indian River Lagoon and adjacent offshore coastal waters of Florida in 2004 | 5.60 | 66.4 | 10.4 | 10.9 | 1.68 | 2.80 | 2.24 | 0.03 | [16] |
| BF.U4 | Red drum muscle, n=11, Samples were collected from estuarine waters of the Indian River Lagoon and adjacent offshore coastal waters of Florida in 2004 | 4.59 | 66.4 | 11.9 | 11.7 | 2.09 | 3.34 | 0.00 | 0.02 | [16] |
| BF.U5 | Hardhead catfish muscle, n=8, Samples were collected from estuarine waters of the Indian River Lagoon and adjacent offshore coastal waters of Florida in 2004 | 1.69 | 34.3 | 22.8 | 9.61 | 13.6 | 12.1 | 0.05 | 5.84 | [16] |
| BF.U6 | Atlantic stingray muscle, n=7, Samples were collected from estuarine waters of the Indian River Lagoon and adjacent offshore coastal waters of Florida in 2004 | 0.99 | 48.4 | 34.1 | 5.93 | 6.42 | 2.96 | 0.99 | 0.25 | [16] |
| BF.U7 | Spiny dogfish muscle, n=5, Samples were collected from estuarine waters of the Indian River Lagoon and adjacent offshore coastal waters of Florida in 2004 | 1.62 | 30.5 | 7.28 | 8.89 | 1.89 | 4.31 | 0.00 | 45.6 | [16] |
| BF.U8 | Atlantic sharpnose shark muscle, n=5, Samples were collected from estuarine waters of the Indian River Lagoon and adjacent offshore coastal waters of Florida in 2004 | 0.80 | 4.88 | 2.01 | 2.65 | 0.73 | 1.50 | 0.00 | 87.4 | [16] |
| BF.U9 | Bull shark muscle, n=7, collected from coastal waters adjacent to the Indian River Lagoon in 2002-2004 | 0.56 | 29.8 | 0.91 | 9.56 | 2.73 | 8.56 | 0.00 | 47.9 | [16] |
| BM.U1 | Archived blubber samples from bottlenose dolphin collected form eastern (Atlantic Ocean) coast of Florida during 2001-2004 | 0.30 | 63.5 | 6.58 | 16.7 | 3.02 | 7.23 | 2.50 | 0.00 | [16] |
| BM.U2 | Striped dolphin blubber, collected along the west coast of florida | 2.43 | 39.47 | 10.6 | 14.0 | 8.30 | 24.47 | 0.74 | 0.00 | [16] |
| BI.E3 | Shrimp were collected from the Scheldt estuary (The Netherlands) at three different locations in November 2001 | 1.92 | 39.0 | 28.6 | 11.1 | 2.92 | 2.60 | 0.00 | 14.0 | [17] |
| BI.E4 | Hermit crab (abdomen) were sampled between 52 and 58°N and 1°W-10°E, covering a large part of the North Sea basin and the Skagerrak, the connection between the North Sea and the Baltic Sea. in August-September 1999 | 2.77 | 50.3 | 15.9 | 19.1 | 3.29 | 8.67 | 0.00 | 0.00 | [18] |
| BI.E5 | Whelk soft (parts) were sampled between 52 and 58°N and 1°W-10°E, covering a large part of the North Sea basin and the Skagerrak, the connection between the North Sea and the Baltic Sea. in August-September 1999 | 4.31 | 23.7 | 17.2 | 12.1 | 25.0 | 17.7 | 0.00 | 0.00 | [18] |
| BI.E6 | Shrimp (whole body) were sampled between 52 and 58°N and 1°W-10°E, covering a large part of the North Sea basin and the Skagerrak, the connection between the North Sea and the Baltic Sea. in August-September 1999 | 4.68 | 66.7 | 9.19 | 12.4 | 0.00 | 7.03 | 0.00 | 0.00 | [18] |
| BF.E1 | Pike (n=51) were caught in the Lumparn estuary in the Åland archipelago in the Baltic Sea | 1.79 | 63.4 | 5.00 | 19.6 | 1.43 | 7.15 | 0.03 | 1.52 | [19] |
| BF.E2 | Perch (n=120) were caught in the Lumparn estuary in the Åland archipelago in the Baltic Sea | 1.17 | 58.4 | 8.17 | 15.2 | 2.39 | 7.01 | 0.10 | 7.59 | [19] |
| BI.E7 | Roach (n=23) were caught in the Lumparn estuary in the Åland archipelago in the Baltic Sea | 0.43 | 15.5 | 0.15 | 2.66 | 0.14 | 1.36 | 0.02 | 79.78 | [19] |
| BF.E3 | Herring Liver, the herring samples were caught in the Southern Bight in October 2000 | 4.28 | 50.9 | 24.4 | 14.0 | 2.65 | 3.67 | 0.00 | 0.00 | [18] |
| BF.E4 | Herring filet, the herring samples were caught in the Southern Bight in October 2000 | 3.04 | 60.8 | 17.6 | 14.9 | 1.28 | 2.40 | 0.00 | 0.00 | [18] |
| BF.E5 | Cod liver, collected in the North Sea and the Skagerrak | 4.08 | 65.1 | 5.79 | 21.7 | 0.46 | 2.89 | 0.00 | 0.00 | [18] |
| BF.E6 | Cod filet, collected in the North Sea and the Skagerrak | 3.74 | 60.6 | 7.31 | 21.4 | 0.00 | 6.95 | 0.00 | 0.00 | [18] |
| BF.E7 | Whiting liver, in the North Sea and the Skagerrak | 3.23 | 66.3 | 12.5 | 13.4 | 1.08 | 3.41 | 0.00 | 0.00 | [18] |
| BF.E8 | Whiting filet, collected in the North Sea and the Skagerrak | 3.42 | 53.1 | 18.2 | 19.0 | 0.00 | 6.26 | 0.00 | 0.00 | [18] |
| BM.E1 | Harbor porpoise liver, the samples of harbor porpoise originated from the southern North Sea and were obtained from Dr. Chris Smeenk and Drs. Marjan Addink of the Museum of Natural History “Naturalis” in Leiden, The Netherlands | 0.96 | 40.9 | 22.8 | 16.2 | 6.30 | 12.9 | 0.00 | 0.00 | [18] |
| BM.E2 | Harbor porpoise blubber, the samples of harbor porpoise originated from the southern North Sea and were obtained from Dr. Chris Smeenk and Drs. Marjan Addink of the Museum of Natural History “Naturalis” in Leiden, The Netherlands | 1.35 | 51.2 | 22.5 | 14.7 | 3.86 | 6.43 | 0.00 | 0.00 | [18] |
| BM.E3 | Harbor seal liver, the samples of harbor seals were obtained from Dr. Ursula Siebert of the Centre for Research and Technology in Bu¨sum, Germany, and originated from Wadden Sea of Schleswig-Holstein (Germany) | 3.31 | 76.0 | 7.85 | 4.55 | 4.55 | 3.72 | 0.00 | 0.00 | [18] |
| BM.E4 | Harbor seal blubber, the samples of harbor seals were obtained from Dr. Ursula Siebert of the Centre for Research and Technology in Bu¨sum, Germany, and originated from Wadden Sea of Schleswig-Holstein (Germany) | 0.89 | 64.5 | 17.5 | 7.67 | 7.06 | 2.42 | 0.00 | 0.00 | [18] |
| S.E1 | Sediments from six locations in Belgian North Sea (S1-S6) | 0.23 | 1.62 | 2.31 | 0.46 | 0.23 | 0.23 | 0.23 | 94.7 | [20] |
| S.E2 | Coastal sediments, sediment samples were collected from several hot spots on the Spanish coast, such as the harbours of Almeria and Tarragona, and mouths of Besós and Llobregat rivers in Barcelona. | 0.00 | 1.59 | 3.18 | 1.36 | 0.00 | 0.00 | 3.27 | 90.6 | [21] |
| S.E3 | Surface sediments (0-2 cm) was collected at 10 different locations in Danish marine territory | 0.00 | 3.50 | 5.83 | 0.96 | 0.85 | 0.00 | 0.00 | 88.9 | [22] |
| S.E4 | Sampling sites were chosen so that different parts of the Dutch sector of the North Sea continental shelf were covered, in spring of 2000 | 0.14 | 1.92 | 2.15 | 0.14 | 0.32 | 0.35 | 0.00 | 95.0 | [23] |
| S.K1 | Surface sediment (at a depth of 0-4 cm) were sampled at the industrialized bays Ulsan, Busan, Jinhae Bay, from February 2003 to March 2004 | 0.02 | 0.30 | 0.43 | 0.07 | 0.18 | 0.14 | 0.64 | 98.2 | [24] |
| S.K2 | Surface sediments (0-4 cm) were sampled at 25 locations from Korean coast from February to June 2004, with 1km of the coastline | 0.04 | 0.68 | 0.06 | 1.52 | 0.33 | 1.33 | 0.94 | 95.1 | [25] |
| BI.K1 | Mussels (Mytilus coruscus and M. edulis) and oysters (Crassostrea gigas), found on piers, rocks and buoys in the water column, were scraped with a rake during low tide. M.edulis at Stations 6-18, and C.gigas at Stations 19-25, on the basis of availability of each species at the sampling locations. | 0.63 | 12.3 | 3.42 | 2.56 | 0.47 | 1.10 | 0.73 | 78.8 | [25] |
| BZ.E1 | Zooplankton Samples (n=3) were taken with a 90μm mesh size net in the central part of the Baltic Sea in the summer of 1999. | 4.54 | 40.1 | 9.95 | 3.14 | 1.92 | 2.62 | 1.06 | 36.6 | [26] |
| BF.E9 | Sprat (n=6) were caught with gill nets in the central part of the Baltic Sea in the summer and autumn of 1998 | 3.36 | 67.2 | 10.3 | 11.6 | 1.99 | 3.58 | 0.05 | 1.84 | [26] |
| BF.E10 | Herring (n=5) were caught with gill nets in the central part of the Baltic Sea in the summer and autumn of 1998 | 2.53 | 63.3 | 15.5 | 9.70 | 0.23 | 5.34 | 0.06 | 3.38 | [26] |
| BF.E11 | Salmon (n=10) were caught with gill nets in the central part of the Baltic Sea in the summer and autumn of 1998 | 2.21 | 69.5 | 12.6 | 8.85 | 2.43 | 3.00 | 0.02 | 1.30 | [26] |
| BI.J1 | Snails (Cerithidea rhizophorarum, n = 10) were handpicked at a range of approximately 3000 m (salinity 19.7–29.5) from the river mouth of Ariake Sea, Japan during October 2012. | 10.47 | 6.47 | 2.09 | 0.95 | 6.47 | 0.00 | 3.14 | 70.41 | [27] |
| BF.J1 | Spotnape ponyfish (Nuchequula nuchalis, n = 5) were collected using a casting net at a range of approximately 3000 m (salinity 19.7–29.5) from the river mouth of Ariake Sea, Japan, during October 2012. | 35.77 | 39.90 | 6.33 | 3.85 | 1.38 | 4.95 | 1.21 | 6.60 | [27] |
| BF.J2 | Javelin goby (Acanthogobius hasta, n = 3) were collected using a casting net at a range of approximately 3000 m (salinity 19.7–29.5) from the rive rmouth of Ariake Sea, Japan during October 2012. | 21.04 | 48.10 | 11.02 | 4.01 | 2.20 | 5.01 | 4.41 | 4.21 | [27] |
| BF.J3 | Yellowfin goby (Acanthogobius flavimanus, n = 4) were collected using a casting net at a range of approximately 3000 m (salinity 19.7–29.5) from the river mouth of Ariake Sea, Japan, during October 2012. | 14.91 | 51.24 | 21.43 | 6.06 | 0.00 | 5.59 | 0.78 | 0.00 | [27] |
| BF.J4 | Sea bass were collected using a casting net at a range of approximately 3000 m (salinity 19.7–29.5) from the river mouth of Ariake Sea, Japan during October 2012. | 22.50 | 47.50 | 4.08 | 6.08 | 3.33 | 5.00 | 2.33 | 9.17 | [27] |
| BF.J5 | Grey mullet were collected using a casting net at a range of approximately 3000 m (salinity 19.7–29.5) from the river mouth of Ariake Sea, Japan during October 2012. | 26.43 | 56.93 | 3.66 | 4.27 | 2.03 | 3.05 | 0.58 | 3.05 | [27] |
| BI.C2 | Mussel (Mytilus edulis) samples were collected from Bo Sea, China, except for sites S1, S2, S3 and S4. | 3.32 | 9.30 | 2.66 | 1.00 | 1.00 | 1.66 | 0.33 | 80.73 | [4] |
| BI.C3 | Clam from a heavily contaminated site Yuandang Lagoon.Xia Men, southeast of China | 1.59 | 0.79 | 0.79 | 0.00 | 5.56 | 1.59 | 8.73 | 80.95 | [7] |
| BI.C4 | Crab (n=5) from a heavily contaminated site Yuandang Lagoon, Xia Men, southeast of China | 1.94 | 1.94 | 0.00 | 0.00 | 1.94 | 1.94 | 7.77 | 84.47 | [7] |
| BF.C2 | Sea-bass (n=6) from a heavily contaminated site Yuandang Lagoon, Xia Men, southeast of China | 6.76 | 51.35 | 8.11 | 9.46 | 10.81 | 9.46 | 4.05 | 0.00 | [7] |
| BF.C3 | Tilapia (n=7) from a heavily contaminated site Yuandang Lagoon, Xia Men, southeast of China | 2.56 | 41.03 | 7.69 | 17.95 | 12.82 | 10.26 | 7.69 | 0.00 | [7] |
| BF.C4 | Banded grouper (n=3) from a heavily contaminated site Yuandang Lagoon, Xia Men, southeast of China | 3.03 | 42.42 | 9.09 | 15.15 | 12.12 | 6.06 | 12.12 | 0.00 | [7] |
| BF.C5 | Houttuyn (n=5) from a heavily contaminated site Yuandang Lagoon, Xia Men, southeast of China | 29.85 | 41.79 | 2.99 | 8.96 | 4.48 | 7.46 | 4.48 | 0.00 | [7] |
| BF.C6 | Mullet (n=5) from a heavily contaminated site Yuandang Lagoon, Xia Men, southeast of China | 23.73 | 44.07 | 8.47 | 10.17 | 3.39 | 5.08 | 5.08 | 0.00 | [7] |
| BF.C7 | Chinese elops (n=5) from a heavily contaminated site Yuandang Lagoon, Xia Men, southeast of China the herring samples were caught in the Southern Bight in October 2000 | 12.31 | 55.38 | 6.15 | 13.85 | 4.62 | 0.00 | 4.62 | 0.00 | [7] |
| BF.C8 | Yellow fin sea-bream (n=5) from a heavily contaminated site Yuandang Lagoon, Xia Men, southeast of China the herring samples were caught in the Southern Bight in October 2000 | 19.35 | 43.55 | 9.68 | 12.90 | 4.84 | 4.84 | 4.84 | 0.00 | [7] |
| BF.C9 | Black sea-bream (n=5) from a heavily contaminated site Yuandang Lagoon, Xia Men, southeast of China | 8.45 | 49.30 | 7.04 | 12.68 | 11.27 | 7.04 | 4.23 | 0.00 | [7] |
| BF.U10 | Whitecroaker (n=6 composite samples, including Oakland, South Bay, and San Pablo Bay, each site has two composite, five fish per composite) were collected in 2006 from popular recreational fishing sites in three Bay segments. In the San Francisco Estuary, US), median value is calculated, data below detection limit, were treated as half of the detection limit, 28=28/33 | 0.84 | 70.77 | 0.96 | 23.20 | 0.33 | 3.84 | 0.05 | 0.00 | [28] |
| BF.U11 | Shiner surfperch (n=8 composite samples, each site has two composite, 20 fish per composite) were collected in 2006 from popular recreational fishing sites in three Bay segments, in the San Francisco Estuary, US. data below detection limit, were treated as half of the detection limit, 28=28/33 | 1.80 | 71.99 | 5.15 | 16.98 | 0.44 | 3.47 | 0.16 | 0.00 | [28] |
| BM.U3 | San Francisco Bay four female harbor seal blubber, were sampled, in the Central Bay segment of San Francisco Bay, US. in 2007 and 2008, data below detection limit, were treated as half of the detection limit,28=28/33 | 0.46 | 82.27 | 8.37 | 5.94 | 1.61 | 1.35 | 0.00 | 0.00 | [28] |
| BM.U4 | San Francisco Bay one adult male harbor seal blubber, were sampled, in the Central Bay segment of San Francisco Bay, US in 2007 and 2008, data below detection limit, were treated as half of the detection limit,28=28/33 | 0.25 | 73.65 | 6.50 | 4.27 | 10.37 | 4.93 | 0.03 | 0.00 | [28] |
| BM.U5 | San Francisco Bay 12 pups harbor seal blubber were sampled, in the Central Bay segment of San Francisco Bay, US in 2007 and 2008, data below detection limit, were treated as half of the detection limit, 28=28/33 | 0.60 | 84.01 | 7.57 | 5.01 | 1.86 | 0.94 | 0.01 | 0.00 | [28] |
| BI.J2 | Oyster (Crassostrea sp.) were collected from Tokyo Bay in Japanese coastal waters. Whole soft tissues of these samples were pooled, homogenized, and analysis | 10.13 | 51.33 | 11.84 | 8.16 | 1.11 | 2.48 | 0.00 | 14.95 | [29] |
| BI.J3 | Oyster (Crassostrea sp.) were collected from Minamata Bay in Japanese coastal waters. Whole soft tissues of these samples were pooled, homogenized, and analysis. | 17.14 | 69.49 | 7.66 | 4.13 | 0.01 | 1.57 | 0.00 | 0.00 | [29] |
| BZ.E2 | Calanus were sampled in the southern Oslofjord (Hvaler: 59° N, 11° E), Norway | 1.59 | 46.75 | 35.36 | 7.97 | 3.47 | 2.49 | 0.77 | 1.59 | [30] |
| BF.E12 | Atlantic cod were sampled in the southern Oslofjord (Hvaler: 59° N, 11° E), Norway | 2.47 | 70.29 | 2.17 | 14.17 | 5.32 | 2.39 | 0.73 | 2.47 | [30] |
| BM.E5 | Harbor seal were sampled in the southern Oslofjord (Hvaler: 59° N, 11° E), Norway | 0.32 | 60.73 | 18.64 | 5.26 | 9.97 | 4.76 | 0.00 | 0.32 | [30] |
| BZ.E3 | Calanus finmarchicus were sampled at Froan in the Norwegian Sea on the west coast of Norway (64° N, 9° E) | 0.00 | 69.57 | 18.26 | 12.17 | 0.00 | 0.00 | 0.00 | 0.00 | [30] |
| BF.E13 | Atlantic cod were sampled at Froan in the Norwegian Sea on the west coast of Norway (64° N, 9° E) | 4.27 | 70.37 | 1.60 | 10.67 | 3.83 | 3.83 | 1.17 | 4.27 | [30] |
| BM.E6 | Harbor seal were sampled at Froan in the Norwegian Sea on the west coast of Norway (64° N, 9° E) | 0.69 | 82.56 | 8.13 | 3.43 | 3.75 | 0.66 | 0.10 | 0.69 | [30] |
| BF.E14 | Polar cod were sampled at Bear Island in the Barents Sea (74° 27’N, 19°E) | 3.68 | 73.06 | 0.00 | 15.20 | 0.00 | 4.37 | 0.00 | 3.68 | [30] |
| BZ.E4 | Calanus glacialisa were sampled from Spitsbergen, Norway | 0.00 | 50.00 | 50.00 | 0.00 | 0.00 | 0.00 | 0.00 | 0.00 | [30] |
| BM.E7 | Harbor seal were sampled from Spitsbergen, Norway | 0.83 | 81.95 | 4.51 | 2.49 | 5.71 | 0.97 | 2.72 | 0.83 | [30] |
| BI.K2 | Bivalves like Mussels (Mytilus coruscus and M. edulis) and oysters (Crassostrea gigas), found on piers, rocks, and buoys in the water column, were scraped with a rake during low tide. found on piers, rocks, and buoys in the water column, were scraped with a rake during low tide along Korean costal | 0.63 | 12.33 | 3.42 | 2.56 | 0.47 | 1.10 | 0.73 | 78.76 | [25] |
| BF.C10 | Large yellow croaker, n=13, were collected in April and August of 2004 from the Pearl River Estuary, South of China | 0.88 | 23.28 | 3.22 | 5.24 | 1.19 | 2.08 | 0.37 | 63.72 | [31] |
| BF.C11 | Silvery pomfret, n=10, were collected in April and August of 2004 from the Pearl River Estuary, South of China | 4.11 | 55.30 | 12.15 | 8.03 | 1.26 | 2.11 | 0.00 | 17.04 | [31] |
| BF.C12 | Flathead fish, n=17, were collected in April and August of 2004 from the Pearl River Estuary, South of China | 2.76 | 66.69 | 5.25 | 15.95 | 2.56 | 6.78 | 0.00 | 0.00 | [31] |
| BF.C13 | Robust tongue fish, n=8 were collected in April and August of 2004 from the Pearl River Estuary, South of China | 1.02 | 56.73 | 24.52 | 9.56 | 2.72 | 5.18 | 0.26 | 0.00 | [31] |
| BI.C5 | Jinga shrimp, n=10 were collected in April and August of 2004 from the Pearl River Estuary, South of China | 2.21 | 60.01 | 26.68 | 6.14 | 2.51 | 1.83 | 0.62 | 0.00 | [31] |
| BI.C6 | Greasy-back shrimp, n=10 were collected in April and August of 2004 from the Pearl River Estuary, South of China edian | 2.40 | 43.04 | 15.45 | 3.32 | 1.90 | 1.79 | 0.99 | 31.11 | [31] |
| BI.C7 | Mantis shrimp, n=9, were collected in April and August of 2004 from the Pearl River Estuary, South of China | 3.01 | 52.50 | 8.57 | 11.09 | 1.01 | 1.21 | 0.00 | 22.62 | [31] |

1 Not detected are treated as 0

**References of Supporting Information:**

[1] G. M. LaA, R. C. Hale, E. Harvey, "Detailed polybrominated diphenyl ether (PBDE) congener composition of the widely used penta-, octa-, and deca-PBDE technical flame-retardant mixtures," *Environmental Science & Technology*, vol. 40, no. 20, pp. 6247-6254, 2006.

[2] B. X. Mai, S. J. Chen, X. J. Luo, et al, "Distribution of polybrominated diphenyl ethers in sediments of the Pearl River Delta and adjacent South China Sea," *Environmental Science & Technology*, vol. 39, no. 10, pp. 3521-3527, 2005.

[3] Y. Liu, G. J. Zheng, H. Yu, et al, "Polybrominated diphenyl ethers (PBDEs) in sediments and mussel tissues from Hong Kong marine waters," *Marine Pollution Bulletin*, vol. 50, no. 11, pp. 1173-1184, 2005.

[4] Z. Wang, X. Ma, Z. Lin, G. Na, Z. Yao, "Congener specific distributions of polybrominated diphenyl ethers (PBDEs) in sediment and mussel (Mytilus edulis) of the Bo Sea, China," *Chemosphere*, vol. 74, no. 7, pp. 896-901, 2009.

[5] L. Liu, H. Li, Z. Wang, R. Liu, Y. Zhang, K. Lin, "Insights into spatially and temporally co-occurring polybrominated diphenyl ethers in sediments of the East China Sea," *Chemosphere*, vol. 123, no. 0, pp. 55-63, 2015.

[6] X. Zhao, H. Zhang, Y. Ni, et al, "Polybrominated diphenyl ethers in sediments of the Daliao River Estuary, China: Levels, distribution and their influencing factors," *Chemosphere*, vol. 82, no. 9, pp. 1262-1267, 2011.

[7] Q. Li, C. Yan, Z. Luo, X. Zhang, "Occurrence and levels of polybrominated diphenyl ethers (PBDEs) in recent sediments and marine organisms from Xiamen offshore areas, China," *Marine Pollution Bulletin*, vol. 60, no. 3, pp. 464-469, 2010.

[8] X. Pan, J. Tang, J. Li, G. Zhong, Y. Chen, G. Zhang, "√Polybrominated diphenyl ethers (PBDEs) in the riverine and marine sediments of the Laizhou Bay area, North China," *Journal of Environmental Monitoring*, vol. 13, no. 4, pp. 886-893, 2011.

[9] C. Marvin, J. Waltho, J. Jia, D. Burniston, "Spatial distributions and temporal trends in polybrominated diphenyl ethers in Detroit River suspended sediments," *Chemosphere*, vol. 91, no. 6, pp. 778-783, 2013.

[10] Z. Cheng, T. Lin, W. Xu, et al, "A preliminary assessment of polychlorinated biphenyls and polybrominated diphenyl ethers in deep-sea sediments from the Indian Ocean," *Marine Pollution Bulletin*, vol., no. 0, pp.

[11] C. S. Kwan, H. Takada, R. Boonyatumanond, et al, "Historical occurrences of polybrominated diphenyl ethers and polychlorinated biphenyls in Manila Bay, Philippines, and in the upper Gulf of Thailand," *Science of the Total Environment*, vol. 470, pp. 427-437, 2014.

[12] J. Peng, C. Huang, Y. Weng, H. Yak, "Determination of polybrominated diphenyl ethers (PBDEs) in fish samples from rivers and estuaries in Taiwan," *Chemosphere*, vol. 66, no. 10, pp. 1990-1997, 2007.

[13] L. G. Chen, B. X. Mai, X. H. Bi, et al, "Concentration levels, compositional profiles, and gas-particle partitioning of polybrominated diphenyl ethers in the atmosphere of an urban city in South China," *Environmental Science & Technology*, vol. 40, no. 4, pp. 1190-1196, 2006.

[14] B. Strandberg, N. G. Dodder, I. Basu, R. A. Hites, "Concentrations and spatial variations of polybrominated diphenyl ethers and other organohalogen compounds in Great Lakes air," *Environmental Science & Technology*, vol. 35, no. 6, pp. 1078-1083, 2001.

[15] M. Yang, H. Qi, H. Jia, et al, "Polybrominated Diphenyl Ethers in Air across China:Levels, Compositions, and Gas-Particle Partitioning," *Environmental Science & Technology*, vol., no. 47, pp. 8978-8984, 2013.

[16] B. Johnson-Restrepo, K. Kannan, R. Addink, D. H. Adams, "Polybrominated diphenyl ethers and polychlorinated biphenyls in a marine foodweb of coastal Florida," *Environmental Science & Technology*, vol. 39, no. 21, pp. 8243-8250, 2005.

[17] T. A. Verslycke, A. D. Vethaak, K. Arijs, C. R. Janssen, "Flame retardants, surfactants and organotins in sediment and mysid shrimp of the Scheldt estuary (The Netherlands)," *Environmental Pollution*, vol. 136, no. 1, pp. 19-31, 2005.

[18] J. P. Boon, W. E. Lewis, M. R. Tjoen-A-Choy, et al, "Levels of polybrominated diphenyl ether (PBDE) flame retardants in animals representing different trophic levels of the North Sea food Web," *Environmental Science & Technology*, vol. 36, no. 19, pp. 4025-4032, 2002.

[19] S. Burreau, Y. Zebuhr, D. Broman, R. Ishaq, "Biomagnification of polychlorinated biphenyls (PCBs) and polybrominated diphenyl ethers (PBDEs) studied in pike (Esox lucius), perch (Perca fluviatilis) and roach (Rutilus rutilus) from the Baltic Sea," *Chemosphere*, vol. 55, no. 7, pp. 1043-1052, 2004.

[20] S. Voorspoels, A. Covaci, J. Maervoet, P. Schepens, "PBDEs in marine and freshwater sediments from Belgium: levels, profiles and relations with biota," *Journal of Environmental Monitoring*, vol. 6, no. 11, pp. 914-918, 2004.

[21] E. Eljarrat, A. De La Cal, D. Larrazabal, et al, "Occurrence of polybrominated diphenylethers, polychlorinated dibenzo-p-dioxins, dibenzofurans and biphenyls in coastal sediments from Spain," *Environmental Pollution*, vol. 136, no. 3, pp. 493-501, 2005.

[22] J. H. Christensen and J. Platz, "Screening of polybrominated diphenyl ethers in blue mussels, marine and freshwater sediments in Denmark," *J Environ Monit*, vol. 3, no. 5, pp. 543-547, 2001.

[23] H. J. Klamer, P. E. Leonards, M. H. Lamoree, L. A. Villerius, J. E. Akerman, J. F. Bakker, "A chemical and toxicological profile of Dutch North Sea surface sediments," *Chemosphere*, vol. 58, no. 11, pp. 1579-1587, 2005.

[24] H. Moon, K. Kannan, M. Choi, H. Choi, "Polybrominated diphenyl ethers (PBDEs) in marine sediments from industrialized bays of Korea," *Marine Pollution Bulletin*, vol. 54, no. 9, pp. 1402-1412, 2007.

[25] H. Moon, K. Kannan, S. Lee, M. Choi, "Polybrominated diphenyl ethers (PBDEs) in sediment and bivalves from Korean coastal waters," *Chemosphere*, vol. 66, no. 2, pp. 243-251, 2007.

[26] S. Burreau, Y. Zebühr, D. Broman, R. Ishaq, "Biomagnification of PBDEs and PCBs in food webs from the Baltic Sea and the northern Atlantic Ocean," *Science of the Total Environment*, vol. 366, no. 2-3, pp. 659-672, 2006.

[27] J. Kobayashi, Y. Imuta, T. Komorita, et al, "Trophic magnification of polychlorinated biphenyls and polybrominated diphenyl ethers in an estuarine food web of the Ariake Sea, Japan," *Chemosphere*, vol. 118, pp. 201-206, 2015.

[28] S. L. Klosterhaus, H. M. Stapleton, M. J. La Guardia, D. J. Greig, "Brominated and chlorinated flame retardants in San Francisco Bay sediments and wildlife," *Environment International*, vol. 47, pp. 56-65, 2012.

[29] D. Ueno, T. Isobe, K. Ramu, et al, "Spatial distribution of hexabromocyclododecanes (HBCDs), polybrominated diphenyl ethers (PBDEs) and organochlorines in bivalves from Japanese coastal waters," *Chemosphere*, vol. 78, no. 10, pp. 1213-1219, 2010.

[30] B. M. Jenssen, E. G. Sormo, K. Baek, et al, "Brominated Flame Retardants in North-East Atlantic Marine Ecosystems," *Environmental Health Perspectives*, vol. 115, pp. 35-41, 2007.

[31] C. Xiang, X. Luo, S. Chen, M. Yu, B. Mai, E. Y. Zeng, "Polybrominated diphenyl ethers in biota and sediments of the Pearl River Estuary, South China," *Environmental Toxicology and Chemistry*, vol. 26, no. 4, pp. 616-623, 2007.
